# Embryonic microglia influence developing hypothalamic glial populations

**DOI:** 10.1186/s12974-020-01811-7

**Published:** 2020-05-06

**Authors:** Candace M. Marsters, Dinushan Nesan, Rena Far, Natalia Klenin, Quentin J. Pittman, Deborah M. Kurrasch

**Affiliations:** 1 Department of Medical Genetics, Cumming School of Medicine, University of Calgary, Calgary, Alberta Canada; 2 Department of Physiology & Pharmacology, Cumming School of Medicine, University of Calgary, Calgary, Alberta Canada; 3 Hotchkiss Brain Institute, University of Calgary, Calgary, Alberta Canada; 4 Alberta Children’s Hospital Research Institute, University of Calgary, Calgary, Alberta Canada

**Keywords:** Neural development, Microglia, Oligodendrocytes, Astrocytes, Hypothalamus, Neural progenitor cells, Oligodendrocyte progenitor cells, Cytokines, Chemokines

## Abstract

**Background:**

Although historically microglia were thought to be immature in the fetal brain, evidence of purposeful interactions between these immune cells and nearby neural progenitors is becoming established. Here, we examined the influence of embryonic microglia on gliogenesis within the developing tuberal hypothalamus, a region later important for energy balance, reproduction, and thermoregulation.

**Methods:**

We used immunohistochemistry to quantify the location and numbers of glial cells in the embryonic brain (E13.5–E17.5), as well as a pharmacological approach (i.e., PLX5622) to knock down fetal microglia. We also conducted cytokine and chemokine analyses on embryonic brains in the presence or absence of microglia, and a neurosphere assay to test the effects of the altered cytokines on hypothalamic progenitor behaviors.

**Results:**

We identified a subpopulation of activated microglia that congregated adjacent to the third ventricle alongside embryonic Olig2+ neural progenitor cells (NPCs) that are destined to give rise to oligodendrocyte and astrocyte populations. In the absence of microglia, we observed an increase in Olig2+ glial progenitor cells that remained at the ventricle by E17.5 and a concomitant decrease of these Olig2+ cells in the mantle zone, indicative of a delay in migration of these precursor cells. A further examination of maturing oligodendrocytes in the hypothalamic grey and white matter area in the absence of microglia revealed migrating oligodendrocyte progenitor cells (OPCs) within the grey matter at E17.5, a time point when OPCs begin to slow their migration. Finally, quantification of cytokine and chemokine signaling in ex vivo E15.5 hypothalamic cultures +/− microglia revealed decreases in the protein levels of several cytokines in the absence of microglia. We assayed the influence of two downregulated cytokines (CCL2 and CXCL10) on neurosphere-forming capacity and lineage commitment of hypothalamic NPCs in culture and showed an increase in NPC proliferation as well as neuronal and oligodendrocyte differentiation.

**Conclusion:**

These data demonstrate that microglia influence gliogenesis in the developing tuberal hypothalamus.

## Background

Microglia are the resident immune cells of the central nervous system (CNS) with well-established roles in surveillance and injury defense in the adult brain. During embryogenesis in the mouse, microglia migrate from the yolk sac into the embryo through newly developed blood vessels around mid-gestation (E9.5) and begin to populate the CNS thereafter [1]. Under non-pathogenic conditions and once the blood brain barrier closes, additional blood-borne monocytes are thought to rarely invade the CNS [2].

Microglia are a specialized cell with distinct RNA signatures from blood monocytes and other tissue macrophages and also across embryonic, postnatal, and adult stages, suggesting unique roles for microglia across temporal windows [3–7]. Indeed, in the postnatal mouse brain, microglia influence dendritic spine formation, synaptic pruning, neuronal connectivity, and behavioral development [8–14]. Moreover, microglia also modulate postnatal neurogenesis in the cortical subventricular zone (SVZ) [15] and hippocampus [16], influence cortical neuronal survival [17], and induce apoptosis and phagocytosis of cleaved caspase 3 (CC3) positive Purkinje cells in the postnatal mouse cerebellum [18]. In contrast, in the fetal brain, microglia align themselves adjacent to NPCs (neural progenitor cells) and promote their differentiation into intermediate precursor cells [19, 20]. Microglia are shown to be involved in maintaining healthy NPC populations in the embryonic cortex and actively engulf NPCs within cortical proliferative zones as a mechanism to eliminate NPCs at the termination of neurogenesis [21, 22]. Furthermore, the elimination of microglia throughout neural development via pharmacological inhibition of Colony stimulating factor 1 receptor (Csf1R) causes neurological deficits, including defects in gross brain architecture and altered neuronal cell number [23, 24], as well as sexually-dimorphic changes in behavior [25]. Finally, genetic ablation of microglia in the embryonic brain reveals defects in outgrowth of forebrain dopaminergic axons [26]. Together, microglia influence both embryonic and postnatal CNS development, with their functional roles varying between regions and time.

In contrast to neuronal development, the influence of microglia on embryonic gliogenesis is poorly understood. In the postnatal brain, microglia can modulate the progression of gliogenesis by interacting with nearby progenitor cells and thereby amplifying oligodendrogenesis in the perinatal SVZ [27]. Additionally, in vitro microglia can release cytokines, namely IL-1β, IL-6, TNFα, and PDGF, that influence the survival and maturation of oligodendrocytes [27, 28]. Other in vitro studies demonstrate that activated microglia may induce astrocyte differentiation through IL-6 [29], as well as oligodendrocyte proliferation and differentiation through IGF-1 [30]. Beyond these positive influences, microglia can also disrupt gliogenesis. For example, microglia can inhibit oligodendrocyte precursor (OPC) survival and proliferation [31] as well as release cytokines and chemokines that regulate NPCs [32] at the expense of developing glial cells [33]. Combined, these data suggest a role for microglial cytokines in influencing gliogenesis.

Here, we examined whether microglia influenced gliogenesis in the embryonic tuberal hypothalamus, a region that contains the energy balance centers of the arcuate nucleus, ventromedial, and dorsomedial hypothalamus [34]. During development, a shared neural progenitor pool lines the third ventricle and sequentially gives rise to neurons (E10–E15.5), glia (E14.5-postnatally), ependymal cells (E16.5-postnatally), and tanycytes (E17.5-postnatally) that populate the mature hypothalamus [35, 36]. It is also one of the few adult brain areas that exhibits adult stem cell properties [37–40]. In this study, we revealed that microglia influenced the progression of embryonic gliogenesis in the developing tuberal hypothalamus, potentially by releasing cytokines that interact with multipotent embryonic NPCs during key windows of glial development.

## Methods

### Mouse strains

Wildtype CD1 mice (Charles River, Strain Code 022) were used for experiments. Mice were held under specified pathogen free conditions and bred to acquire timed-pregnant dams for obtaining embryonic tissue samples. For embryonic staging, female mice were plug checked in mornings, and those with a positive vaginal plug were assigned embryonic day (*E*) 0.5. For postnatal staging, the day of birth was assigned as postnatal day (*P*) 0. For our microglia knockdown model, pregnant dams were given the Csf1R antagonist, PLX5622 (1200 PPM rodent diet, ~ 200 mg/kg dose; Plexxikon), in standard chow (AIN-76A, Open Source Diets) ad lib starting at E3.5 onwards (Supplementary Fig. 1). Pregnant dams were on a 12 h light/dark cycle and housed singly. Samples were taken from a minimum of two separate litters. Animal protocols were approved by the University of Calgary Animal Care Committee and follow the Guidelines of the Canadian Council of Animal Care.

### Fixed tissue preparation

For sample preparation, gravid females were anaesthetized with isoflurane and immediately decapitated. Embryos were removed, and embryonic brains were extracted for E15.5 and E17.5 time points, while whole embryonic heads were taken for E11.5 and E13.5 time points. P2 pups were decapitated directly and brains extracted. Samples were fixed overnight with 4% paraformaldehyde in phosphate buffered saline (PBS), washed in PBS, and then treated with 20% sucrose before being embedded in O.C.T. Brain samples were cryosectioned and put directly onto slides at 10 μm sectioning width with a selection sampling fraction of 1 for every 8 serial sections within our region of interest.

### Immunofluorescence

Brain sections were treated overnight with primary antibody in 5% normal donkey serum/PBS with 0.1% Tween-20 at 4 °C and followed by the appropriate fluorescently conjugated secondary antibody. For samples requiring further unmasking of the epitope, which included anti-Aldh1L1, antigen retrieval was performed by boiling in 6 M sodium citrate buffer at pH 6.0 for 20 min. Slides were then cooled to room temperature and washed 3× with PBS and 0.1% Tween-20, and the standard immunostaining protocol was followed. Well characterized primary antibodies were as follows: rabbit anti-Iba1 (1:500, WAKO), goat anti-Iba1 (Abcam; 1:300), rat anti-SF1 (1:800, kindly provided by Dr. Taro Tachibana, Osaka City University JAPAN), mouse anti-Olig2 (1:300, Millipore), rat anti-LAMP1 (1:800, Millipore), rat anti-CD68 (1:500, BIORAD), goat anti-Sox9 (1:60, R&D systems), rabbit anti-NKX2.1 (1:400, Santa Cruz), sheep anti-Csf1R (1:300, R&D Systems), goat anti-PdgfR alpha (1:150, R&D Systems), rabbit anti-GFAP (1:500; DAKO), rabbit anti-SB100 (1:400; DAKO), and rabbit anti-Aldh1l1 (1:500, Abcam). All appropriate secondary antibodies were Donkey anti-IgG Alexa Fluor conjugated (1:400, ThermoFisher Scientific). All samples were counterstained with Hoechst nuclear stain (1:1000; ThermoFisher Scientific H3570).

### In situ hybridization

In situ DIG labeled riboprobe was made following the DIG RNA labeling kit protocol (Roche) generated from an IMAGE consortium (Lawrence Livermore National Laboratory) cDNA clone: *csf1r* (GenBank BC043054, Mammalian Gene Collection, NIH) in the *pYX-ASC* plasmid vector. In situ hybridization was performed as previously described [41] and following the manufacturer’s protocol accompanying the digoxigenin (DIG)-RNA labeling kit (Roche) and using RNAase free equipment. In short, brain sections were hybridized overnight at 64 °C, washed in 50% formamide/1 × SSC/0.1% Tween-20 at 65 °C, and rinsed in 1 × MABT. Brain sections were incubated overnight at room temperature in diluted alkaline phosphatase (AP)-conjugated anti-DIG antibody (1:2500; Roche) in blocking solution, equilibrated in developing buffer (0.1 M Tris, pH 9.5, 0.1 M NaCl, 0.05 M MgCl2, 0.1% Tween-20, levamisole 0.05%) and developed in NBT + BCIP solution (Roche).

### Imaging, quantification, and statistical analysis

For Iba1+ microglia cell population studies in the tuberal hypothalamus, immunostained embryonic mouse brain slices were scanned using an Olympus VS-110-S5 slide scanner with a UPLSAPO 20X (NA 0.75) objective lens allowing for optical z-stacking of images in the Olympus’ VSW software. Images containing the SF-1+ tuberal hypothalamic area were further acquired, cropped, and flattened from slide scanner images using the Olympus CellSens software and exported as TIFF files. From these images, all Iba1+ microglia cells containing DAPI+ nuclei were manually counted within the entire tuberal hypothalamic region using SF-1, to mark the rostrocaudal length of the hypothalamus and Nxk2.1 to verify the hypothalamic sulcus border at the dorsal edge of the tuberal hypothalamus in adjacent brain sections. Cells were counted at E11.5, E13.5, E15.5, and E17.5 using the Adobe Photoshop CS6 counting software. For volume estimations of the tuberal hypothalamus, the Cavalieri method was used as previously described [42].

For distance to the ventricle, microglia soma centers co-labeled with DAPI were measured to the ventricle edge (protocol adapted from Swinnen et al.) [43]. This number was divided by the total distance of the ventricle edge to the pia edge for each microglia location to provide a percentage of relative distance of microglia from the ventricle when compared to the pia edge. The percentage of the microglia population within each 10% distance interval away from the ventricle was calculated and graphed. Embryonic samples from more than one pregnant dam were used for each experimental group. For other cell counts, images were taken using a Zeiss Axioplan 2 manual compound microscope with a Zeiss Axiocam HRc camera and using the ZENBlue software or a Zeiss LSM700 Confocal microscope, using the ZENBlack software. For quantification, the Adobe Photoshop CS6 counting software was used to manually count cells. Cells were counted from a total of three brain sections per embryo from a minimum of two separate dams in the rostral-to-mid area of the tuberal hypothalamus using SF-1 and Nkx2.1 to mark the rostral edge of the tuberal hypothalamus and the hypothalamic sulcus as the dorsal border, respectively. Statistical differences were assessed using a Student’s *t* test or an ANOVA statistical test with a Tukey post hoc analysis all using the Graphpad Prism 7 and Graphpad Prism 8 software. Dixon’s Q test at 95% confidence was used for criteria to determine outliers.

### Sholl analysis

Protocol was adapted from Derecki et al. [44]. In short, Z-stacked confocal images were taken using a Zeiss LSM700 confocal microscope with a × 40 oil immersion objective. Images were converted to binary using maximum intensity projection, and Sholl analysis was performed using the Image J software with the Sholl analysis plug-in. For each microglia, a starting radius of 10 μm was used with a 5-μm step size. The number of intersections was automatically generated using the software. Microglia located at the ventricle were within a 10% distance from the ventricle when compared to the pia edge, and microglia in the mantle were anywhere between 20 and 90% distance from the pia edge. Microglia cells within the tuberal hypothalamic area from three embryonic brain sections per pup from each of the three different pregnant dams were used for analysis. Statistical differences between the two groups were performed using a non-parametric Mann-Whitney test using the Graphpad Prism 7 software. Total number of intersections from individual microglia was plotted along with bars representing mean ± SD.

### Immunostained area measurements

For CD68 measurements, individual microglia cells were isolated from the ventricle and periphery areas in the rostral tuberal hypothalamus at E15.5 from images taken using a × 20 objective on a Zeiss Axioplan 2 manual compound microscope. Microglia denoted as being located in the ventricular area were within the 10% distance bin of relative distance to the ventricle, and microglia denoted as being located within the periphery were located between the 30 and 90% distance bins of relative distance to the ventricle. The area of CD68 and Iba1 immunostaining was calculated for each image using the measurement tool in ImageJ. CD68 total area was calculated using the area of CD68 within each individual microglia. The CD68 load was calculated by dividing the area of CD68 by the area of Iba1 resulting in a percentage of the area containing CD68 for each individual microglia. A Student’s *t* test was used to determine statistical significance between groups. For PdgfRα+ area coverage calculations, uniform sized images were taken from randomly chosen white matter and grey matter areas in the rostral tuberal hypothalamus at E15.5 and E17.5 using a × 40 objective on a Zeiss Axioplan 2 manual compound microscope. Total area of PdgfRα+ coverage was calculated per image. An ANOVA with Tukey’s post hoc was used to determine statistical significance between treatments and embryonic stages.

### Brain slice culture and analyte analysis

CD1 female mice were bred to acquire timed pregnant dams. Dams were anesthetized, decapitated, and embryo brains extracted at E14.5 embryonic time points. Extracted brains were placed in 4% low melt agarose (Invitrogen) and sliced on a Leica VT1200S vibratome at a thickness of 400 μm in ice cold sterile PBS. Sections containing the third ventricle in the area of the tuberal hypothalamus, as defined by the clear hypothalamic sulcus, were immediately placed on Millicell organotypic slice culture inserts (Millipore) and placed in a cell culture dish with 1 mL of 37 °C culture media containing (v/v): 56.6% DMEM, 28% F-12, 5% Fetal Bovine Serum, 5% Horse Serum, 2% B27, 1% each Glutamax, Penicillin/Streptomycin, and N2 supplement, 0.4% Fungizone. Slices were placed in a humid incubator with ambient oxygen and 5% CO2 at 37 °C. Brain slices were allowed to equilibrate for 24 h after slicing at which point the media was removed, slices were rinsed with fresh 37 °C culture media, and then 1.2 mL of new culture media was added. Cells were then left to culture for 48 h with 0.2 mL of fresh 37 °C culture media added at 24 h. Culture media was collected, spun down at 300 RCF for 10 min, flash frozen, and then was sent for quantification of cytokines using the Mouse Cytokine 32-PLEX Discovery Assay (Millipore, Milliplex) with each sample performed in triplicate. CX3CL1 (Millipore, Milliplex) and CXCL12 (Millipore, Milliplex) were analyzed separately with each sample performed in duplicate. All analytes were evaluated on a Bioplex 200 (Biorad) through Eve Technologies (Eve Technologies, University of Calgary). Samples with measurements below the detection level were extrapolated based on the mathematical formula of the curve when possible or given a value of zero. Statistical differences were assessed using a Welch’s *t* test using the Graphpad Prism 7 software with assumed normality. Dixon’s Q test at 95% confidence was used for criteria to determine outliers.

### Cell culture and neurosphere counts

A previously published neurosphere assay [45] was adapted for use during the window of peak neurogenesis in the mouse hypothalamus [46]. Briefly, pregnant dams were sacrificed on E12.5 and fetuses removed. Two transverse cuts in the fetal brain were made to reveal the developing hypothalamus for microdissection. The tissue was washed in sterile PBS and manually triturated, then spun down at 300 g for 10 min. This step was repeated in NeuroCult™ (Stemcell Technologies, Vancouver) basal medium with 20 ng/mL each of endothelial growth factor and fibroblast growth factor added. Cells were then passed through a 20-μM cell strainer, counted, and plated at 5000 cells/mL in 24-well plates for treatment. Cells were treated with vehicle, CCL2, CCL3, CCL4, CXCL2, and CXCL10, at 10 ng/mL. Cells were incubated for 10 days at 37 °C with 5% CO2, with 50% media replenishment at day 5. At day 10, primary neurospheres were counted and imaged, then dissociated in sterile media, counted, and replated at 1000 cells/mL and cultured again for 10 days as above for secondary neurosphere formation. For differentiation assays, cells were treated with vehicle, CCL2 only, or CXCL10 only at 10 ng/mL. Growth factors were removed, and dissociated cells were allowed to differentiate over 7 days in culture, then fixed in 2% paraformaldehyde in NeuroCult™ media. Fixed cells were analyzed via immunofluorescence for identity as follows: rabbit anti-NeuN (neurons, 1:200, Abcam), mouse anti-Olig2 (oligodendrocytes, 1:400, Millipore), and rabbit anti-Aldh1L1 (astrocytes, 1:500, Abcam).

## Results

### A subpopulation of colonizing microglia congregate near the tuberal hypothalamic third ventricle

**Fig. 1:**
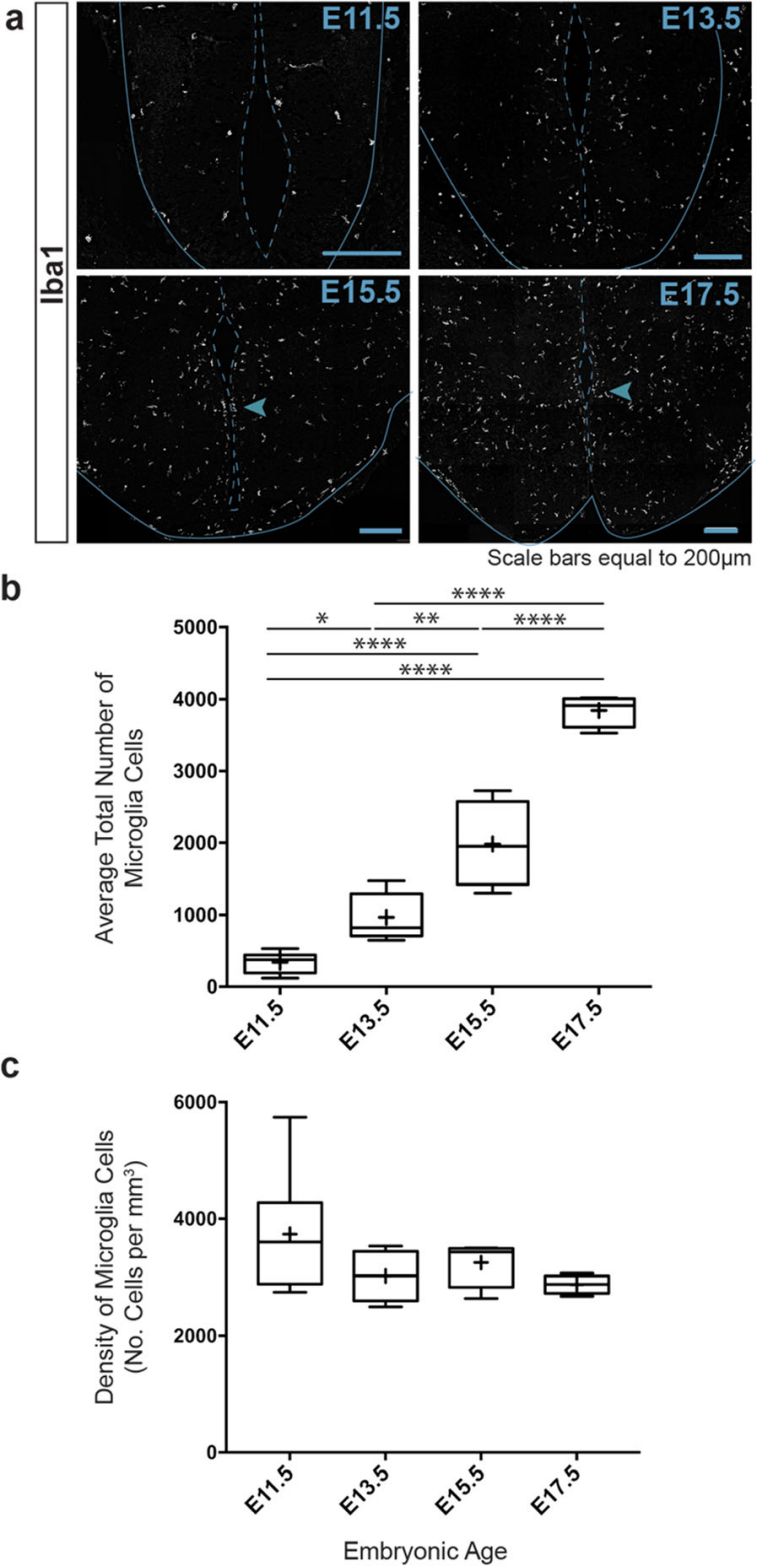
Microglia expand their population during tuberal hypothalamic embryonic development. **a** Representative Iba1 immunostained images of coronal embryonic brain sections within the tuberal hypothalamus at E11.5, E13.5, E15.5, and E17.5 time points. Box and Whisker plots: **b** Total number of Iba1+ microglia within the entire embryonic tuberal hypothalamic area (mean = 339.9 ± 148.2, 965.9 ± 332.0, 1983 ± 599.8, 3845 ± 221.9 cells for E11.5, E13.5, E15.5, and E17.5, respectively; *P* < 0.0001) and (**c**) Density of Iba1+ microglia within the embryonic tuberal hypothalamus (mean = 3335.1 ± 470.6, 3021.6 ± 434.3, 3250.9 ± 410.1, 2875.5 ± 160.0 cells/mm^3^ for E11.5, E13.5, E15.5, and E17.5, respectively; *P* = 0.4616). Samples: *n* = 6, 5, 4, 4 embryos for E11.5, E13.5, E15.5, and E17.5, respectively. Graphs: Box and Whisker plots with middle line representing median, cross representing mean, box extending from the 25th to 75th percentiles, and whiskers at max and min values. Statistics: ANOVA with Tukey post hoc for multiple comparisons, *P ≤ 0.05, **P ≤ 0.01, ****P ≤ 0.0001

Given that microglia colonize the developing brain around E9.5, we first quantified the spatiotemporal invasion and expansion of microglia in the embryonic hypothalamus. We found that microglia invaded the tuberal hypothalamus by E11.5, increasing 10-fold in total numbers between E11.5 and E17.5 (Fig. 1a, b). This expansion was relative to CNS growth since microglial density remained constant between E11.5 and E17.5, demonstrating that as the developing brain enlarged, the number of microglia within the tuberal hypothalamus proportionally increased (Fig. 1c).

**Fig. 2:**
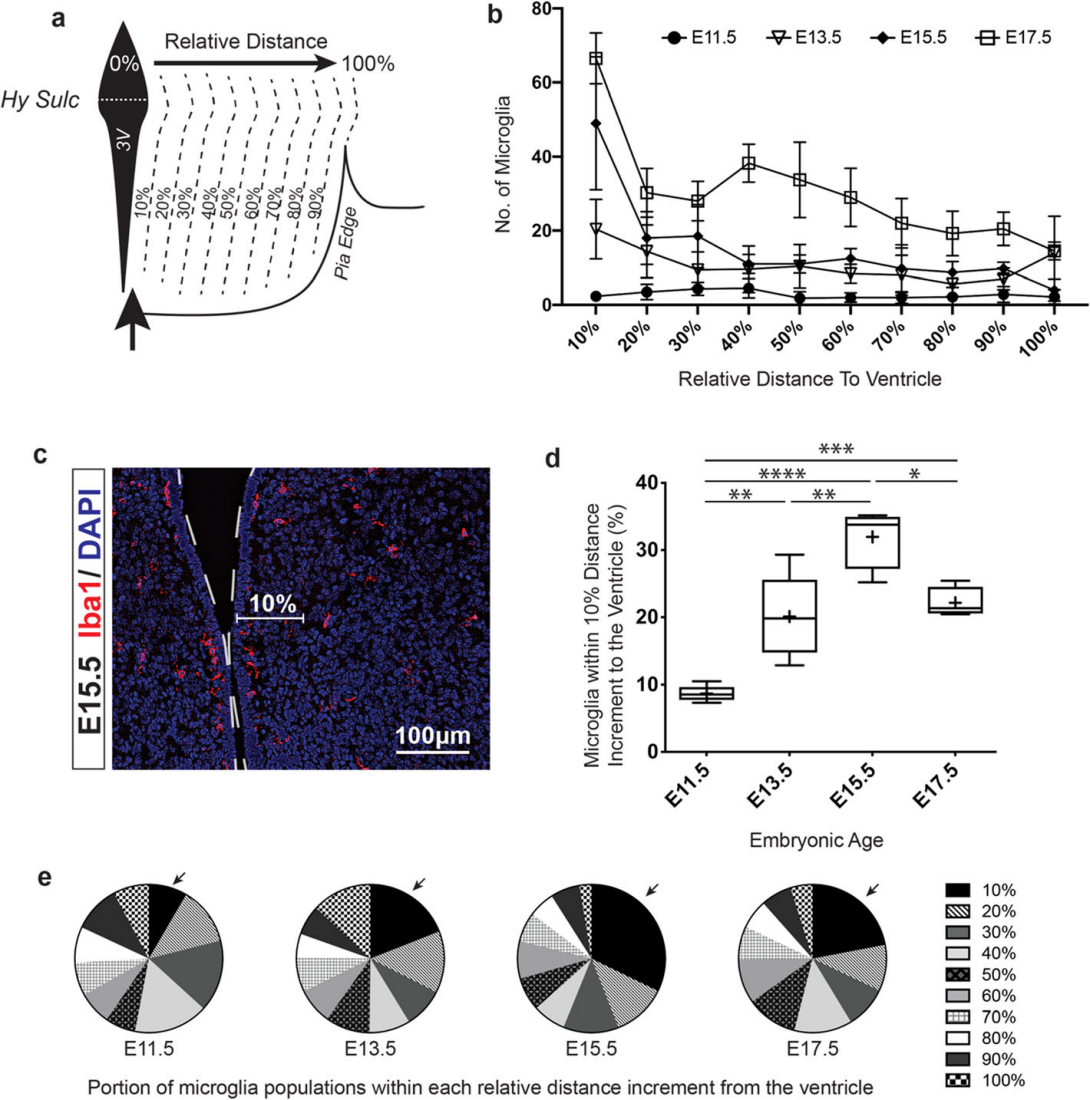
Microglia congregation at the third ventricle peaks at E15.5 in the embryonic tuberal hypothalamus. **a** Illustration depicting the measured relative distances to the third ventricle from the pia edge with a dotted line in the 3V labeling the hypothalamic sulcus (Hy Sulc), the dorsal border of the tuberal hypothalamus. **b** Total microglia population located within each 10% relative increment distance away from the third ventricle at mid- to late-embryonic time points. **c** Cropped Iba1 immunostaining of the third ventricle at E15.5 with the first 10% distance increment highlighted for better visualization. Third ventricle outlined as a dotted white line. **d** Box and Whisker plot representing the percentage of the microglia population that are located within the nearest 10% increment distance adjacent to the third ventricle at E11.5, E13.5, E15.5, and E17.5 embryonic time points (*P* < 0.0001). **e** Parts-of-whole plots showing mean percentage of the microglia population within each 10% relative increment distance away from the third ventricle as part of the total mean population at E11.5 (mean total = 27.7 ± 11.5), E13.5 (mean total = 107.2 ± 45.6), E15.5 (mean total = 152.2 ± 45.7), and E17.5 (mean total = 302.0 ± 40.4). Samples: Counts from the entire tuberal hypothalamic area in *n* = 6, 5, 4, 4 embryos for E11.5, E13.5, E15.5, and E17.5, respectively. Graphs: Parts-of-whole plots showing mean population percentage within each relative distance to the ventricle as part of the total mean population. Box and Whisker plots with middle line representing median, cross representing mean, box extending from the 25th to 75th percentiles, and whiskers at max and min values. Statistics: ANOVA with Tukey post hoc for multiple comparisons, *P ≤ 0.05, **P ≤ 0.01, ***P ≤ 0.001, ****P ≤ 0.0001

We also observed that a subpopulation of microglia congregated along the third ventricle at the later embryonic stages of E15.5 and E17.5 (Fig. 1a, arrows). We quantified this accumulation by binning microglia into 10% intervals starting at the ventricle and progressing outward to the pia edge (measurement method illustrated in Fig. 2a). Starting at E13.5, a larger proportion of microglia were located within the first 10 percentile (i.e., closest to the third ventricle; Fig. 2a arrow) than at other spatial intervals (Fig. 2b). The absolute number of microglia located adjacent the third ventricle increased through E13.5, E15.5, and E17.5 (Fig. 2b), but the highest percentage of the microglia population congregating at the ventricle peaked at E15.5 (Fig. 2c, d, e). Indeed, at this select time point, 32% of the total microglia within the tuberal hypothalamus was in the bin closest to the ventricle, compared to 9%, 20%, and 22% at E11.5, E13.5, and E17.5, respectively (Fig. 2d).

### Microglia that reside adjacent to the third ventricle are more ameboid

**Fig. 3:**
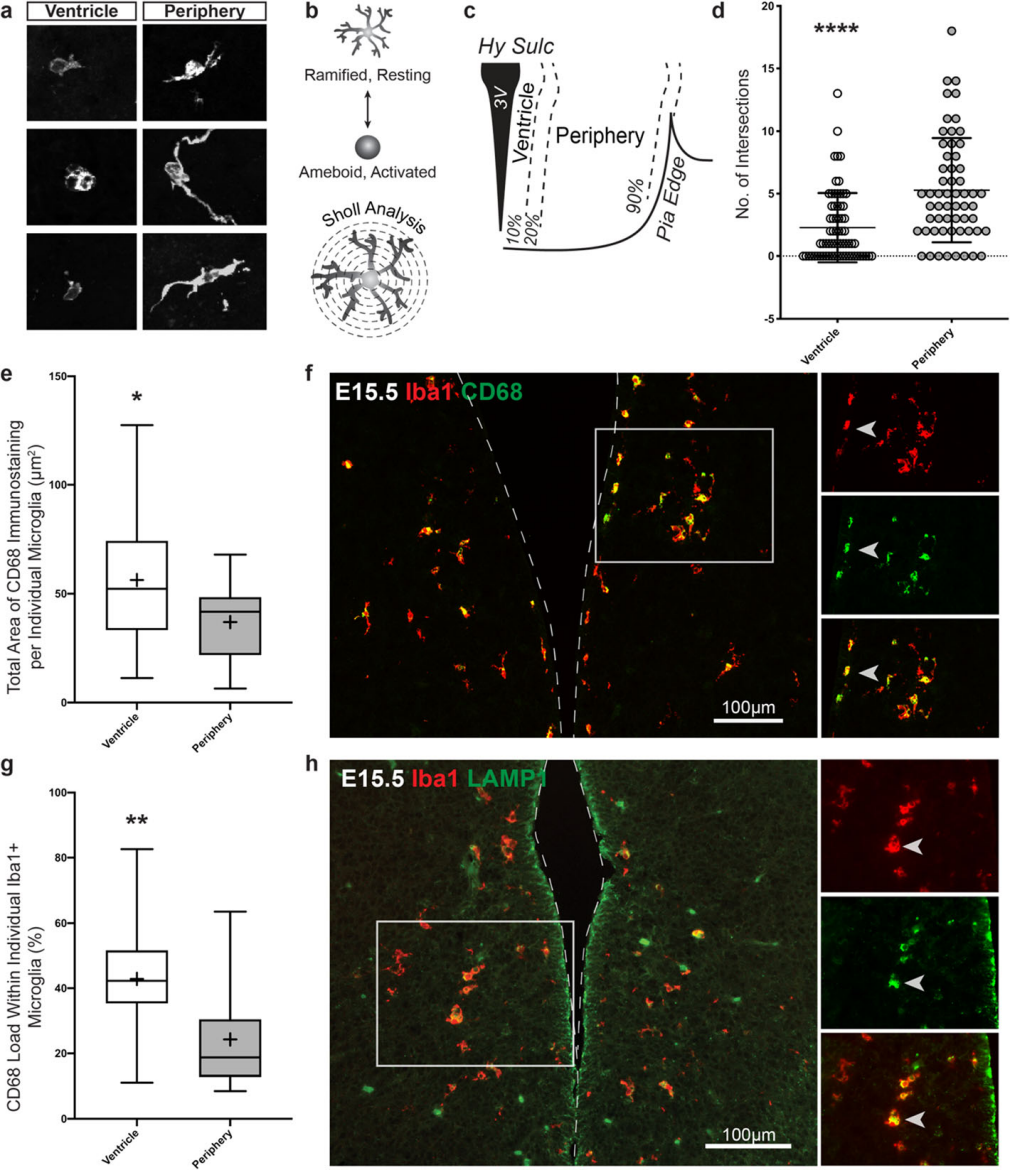
Microglia located near the third ventricle are more ameboid at E15.5. **a** Example phenotypes of E15.5 microglia located near the ventricle and in the periphery. **b** Schematic illustration depicting microglia resting and activated phenotypes and how the concept of Sholl analysis is applied for analyzing microglia processes. **c** Illustration defining the ventricle and periphery area in the tuberal hypothalamus used in the microglia Sholl analysis and CD68 analysis. Ventricle microglia were defined as being localized within a 10% relative distance away from the ventricle whereas peripheral microglia were located at a 30% or further relative distance from the ventricle. **d** Sholl analysis comparing the activation state of E15.5 microglia located near the ventricle (mean value 2.3 ± 2.8 intersections) and in the periphery (mean value 5.3 ± 4.2 intersections). Scatter plot points display individual microglia scores (microglia cells counted within three brain sections from three separate embryos, *n* = 69 cells in ventricle, *n* = 59 cells in periphery; *P* < 0.0001). **e** Total area of CD68 within microglia cells located near the ventricle (mean value = 56.28 ± 30.16 μm^2^) and in the periphery (mean value = 36.91 ± 19.67 μm^2^). **f** Representative image of microglia activation marker, CD68, and Iba1 at E15.5. Left image displays CD68 and Iba1 expression in the tuberal hypothalamus. Right images are of the boxed area near the ventricle showing CD68 co-labeling with Iba1. Arrow points to example of a co-labeled cell. **g** CD68 load within microglia cells located near the ventricle (mean value = 42.86 ± 17.60%) and in the periphery (mean value = 24.23 ± 14.77%; 5 microglia cells analyzed from each area in a rostral tuberal hypothalamic brain section from three separate embryos, *n* = 15 cells in ventricle, 15 cells in periphery; *P* = 0.0464 for CD68 total area and *P* = 0.0039 for CD68 load). **h** Representative image of the lysosomal marker, LAMP-1, and Iba1 at E15.5. Left image shows several Iba1+ microglia co-label with LAMP-1 near the third ventricle. Right images are higher magnifications of boxed area highlighting the co-labeling of Iba1 and LAMP-1. Arrow points to example of a co-labeled cell. Graphs: Scatter plot with mean ± SD. Box and Whisker plots with middle line representing median, cross representing mean, box extending from the 25th to 75th percentiles, and whiskers at max and min values. Statistics: Two-tailed Mann-Whitney *U* test used in Sholl analysis otherwise Student’s *t* test, *P ≤ 0.05, **P ≤ 0.01, ****P ≤ 0.0001

Upon closer examination of the juxta-ventricular residing microglia, we noticed two trends: (1) the microglia were not evenly lined along the dorsoventral axis of the third ventricle but instead were clustered around a specific region ventral to the hypothalamic sulcus (denoted by arrows Fig. 1a) and (2) the microglia appeared larger, more amoeboid, and has less extended processes, particularly at the E15.5 time point (Fig. 3a). To verify that these ventricular microglia indeed were less ramified than peripheral microglia located within the mantle zone, we first employed a Sholl analysis [44] that quantifies microglia morphologies ranging from a ramified/resting state that displays many intersections in a Sholl grid, to an amoeboid/activated state that shows very few or no intersections (Fig. 3b). We sampled microglia that resided in the ventricular zone (binned within the closest 10%) and compared against those localized in the periphery (within the remaining 30–90%; Fig. 3c). We found a significantly lower number of intersections for microglia located near the ventricle compared to those in the periphery at the E15.5 time point (Fig. 3d) suggesting microglia near the ventricle display an ameboid morphology.

Next, we co-immunostained microglia with Iba1 and the activation marker, CD68, to further characterize the state of ventricular microglia at E15.5. CD68 is a lysosomal-associated protein that serves as an activation marker expressed in the monocyte lineage [47], which is also expressed in microglia during embryonic development [48]. We quantified the total area of CD68 immunostaining in Iba1+ microglia cells near the ventricle (Fig. 3e), as defined by the collection of Iba1+ ventricular microglia that displayed extensive CD68 immunostaining within the previously identified clustering region just ventral to the hypothalamic sulcus (Fig. 3f, boxed area). We also quantified the percentage of cell area covered by CD68, indicated as CD68 load, in each microglia cell (Fig. 3g), and compared both of these values with microglia located in the periphery. We found a significantly larger area of CD68 and higher CD68 load in microglia located near the ventricle compared to those in the periphery at the E15.5 time point (Fig. 3e, g), further suggesting that microglia at the ventricle potentially reside in an activated state.

We also examined a second activation marker, LAMP1, which is a sialoglycoprotein found on lysosomal membranes involved in degradation of endocytosed foreign materials during phagocytosis [49]. We found that LAMP1 was expressed in this subset of Iba1+ microglia also in this same region near the third ventricle (Fig. 3h). Combined, these data suggest that microglia near the ventricle at E15.5 reside in a different state than those located in the peripheral mantle zone.

### Microglia congregate near Olig2+ progenitors lining the third ventricle

**Fig. 4:**
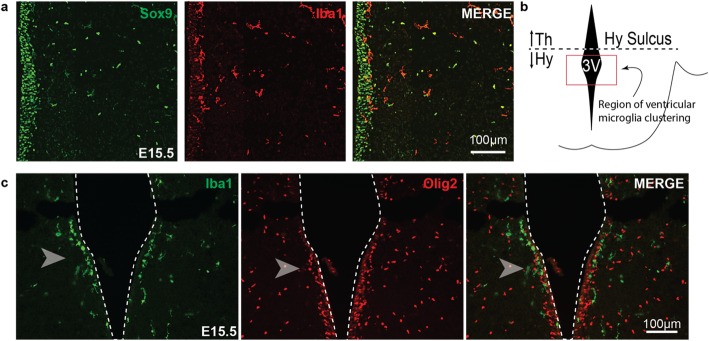
A subset of microglia congregate along the third ventricle near the periventricular Olig2+ neural progenitor cell domain in the mid tuberal hypothalamus at E15.5. **a** Representative image of Iba1 and nuclear cell marker, Sox9, showing Iba1+ microglia in close proximity to periventricular Sox9+ NPCs. **b** Illustration depicting the area imaged with the location at the third ventricle (3V) of the tuberal hypothalamus ventral to the hypothalamic sulcus (Hy Sulc). **c** Representative image of Iba1+ microglia congregation near the Olig2+ domain near the sulcus border of the tuberal hypothalamus. Arrows point to the area of Iba1+ cells in close proximity to the periventricular Olig2+ domain. Third ventricles outlined with dotted white line

Embryonic NPCs line the ventricle and initiate waves of neurogenesis, followed by gliogenesis. In the tuberal hypothalamus, neurogenesis occurs mid-gestation and is completed by E15.5 [36]. This is followed by gliogenesis consisting first of oligodendrogenesis, which begins around E13.5, then astrocytogenesis, which commences at E15.5 [35]. Differentiated neurons/astrocytes and specified OPCs then migrate away from the ventricular zone into the parenchyma where they further mature [38, 39, 50, 51]. Given that a subpopulation of microglia clustered near the third ventricle at E15.5, an area adjacent to where NPCs reside and at a time point consistent with the initiation of gliogenesis in the tuberal hypothalamus, we next explored a role for microglia in glial development. We first examined microglia proximity to NPCs located at the ventricle in the embryonic tuberal hypothalamus. We immunostained E15.5 embryonic brain slices with Iba1 to label microglia and the nuclear neural progenitor marker, Sox9. Sox9 labels embryonic NPCs along the ventricle and glioblasts in the mantle zone [52–55]. We observed a subpopulation of Iba1+ microglia that aligned tightly with Sox9+ NPCs (Fig. 4a) and ventral to the hypothalamic sulcus region (Fig. 4b). We next examined the proximity of Iba1+ microglia to Olig2+ cells that are destined to become glial progenitor cells [56–58] in the ventricular zone at E15.5. We observed Iba1+ microglia were interspersed with Olig2+ progenitors in a region adjacent to the third ventricle, but we noted little evidence of cellular overlap (note the lack of Iba1+/Olig2+ yellow cells) that would be suggestive of appreciable NPC engulfment by microglia (Fig. 4c, arrows).

### Microglia influence the development of tuberal hypothalamic glial populations

Next, we investigated whether these ventricular microglia were involved in the progression of gliogenesis of these Olig2+ cells, which are required for initiation of gliogenesis [35]. To determine whether microglia influenced the progression of glial development in the embryonic hypothalamus, we eliminated microglia using PLX5622 and examined early glioblast populations at: (1) E15.5, the initiation of gliogenesis and the time point when microglia congregate at the ventricle; (2) E17.5, mid-embryonic gliogenesis and following ventricular microglia congregation; and (3) P2, during postnatal gliogenesis and when oligodendrocytes and astrocytes are maturing. To eliminate microglia, we exposed pregnant dams to chow containing PLX5622 (1200 ppm) ad lib starting at E3.5 and for the remainder of the pregnancy. PLX5622 eliminates > 99% of microglia by E15.5 in the tuberal hypothalamus [25], and here we employed a modified paradigm to maintain continued elimination of both Csfr1+ and Iba1+ microglia past E15.5 and into P2 (Supp. Fig. 1).

**Fig. 5:**
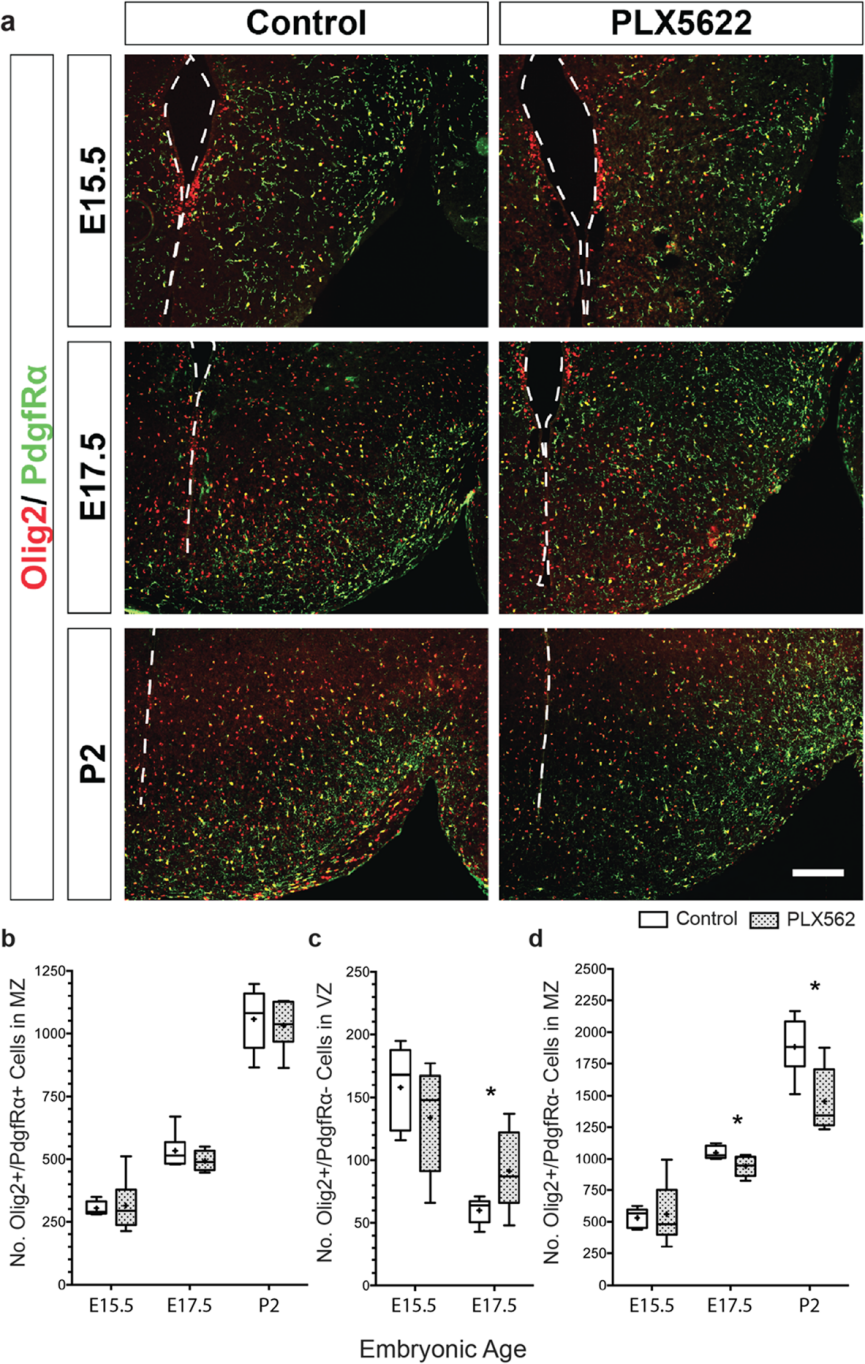
A subset of the Olig2+ cell population in the tuberal hypothalamus is altered in the PLX5622 microglia knock down model. Cell counts from immunostaining in control and PLX5622 treated embryonic and postnatal coronal brain slices in the rostral-to-mid tuberal hypothalamus. **a** Representative images of tuberal hypothalamus immunostained with Olig2/PdgfRα at E15.5, E17.5, and P2 in control and the PLX5622 microglia knock down model. **b** Olig2+/PdgfRα+ cells located within the MZ at E15.5 (*P* = 0.8264), E17.5 (*P* = 0.2609), and P2 (*P* = 0.7102). **c** Olig2+/PdgfRα− cells located adjacent to the ventricle in the VZ region at E15.5 (*P* = 0.3322) and E17.5 (*P* = 0.0457). **d** Olig2+/PdgfRα− cells located within the MZ at E15.5 (*P* = 0.8042), E17.5 (*P* = 0.0163), and P2 (*P* = 0.0119). Samples: Counts combined from three brain slices in embryonic or postnatal brains with *n* = 5, 6 at E15.5; *n* = 6, 6 at E17.5; *n* = 6, 6 at P2 for control and PLX5622, respectively. Graphs: Box and Whisker plots with middle line representing median, cross representing mean, box extending from the 25th to 75th percentiles, and whiskers at max and min values. Statistics: Student’s *t* test, *P ≤ 0.05

To track oligodendrocyte lineage-specified Olig2+ cells (versus unspecified or astrocyte-determined Olig2+ cells), we co-labeled with PdgfRα, a marker for OPCs [59] (Fig. 5a). We counted the number of OPCs (Olig2+/PdgfRα+) in areas adjacent to the ventricular zone and those that had migrated into mantle zone. No difference in the number of Olig2+/PdgfRα+ OPCs was observed between control and PLX5622-exposed developing brains at E15.5, E17.5, or P2 (Fig. 5b). We next determined the number of Olig2+ glial progenitors that had not yet been specified to become oligodendrocytes (Olig2+/PdgfRα−). Here, we measured a significant increase of Olig2+/Pdgfrα− glial progenitor cells located adjacent to the ventricle at E17.5 in the PLX5622 embryonic hypothalami, which coincided with a reduction in the number of Olig2+/PdgfRα− cells in the mantle zone at E17.5 and P2 (Fig. 5c, d). No change in Olig2+/PdgfRα− cells residing at the ventricle or parenchyma was observed at E15.5 (Fig. 5c, d). Combined, an increase in Olig2+/PdgfRα− cells adjacent to the ventricle and a concomitant decrease in this lineage in the mantle zone in our microglia knocked down model suggested that microglia might influence Olig2+ progenitors without affecting the total number of OPCs.

### Microglia influence the phenotype of PdgfRα+ OPCs

**Fig. 6:**
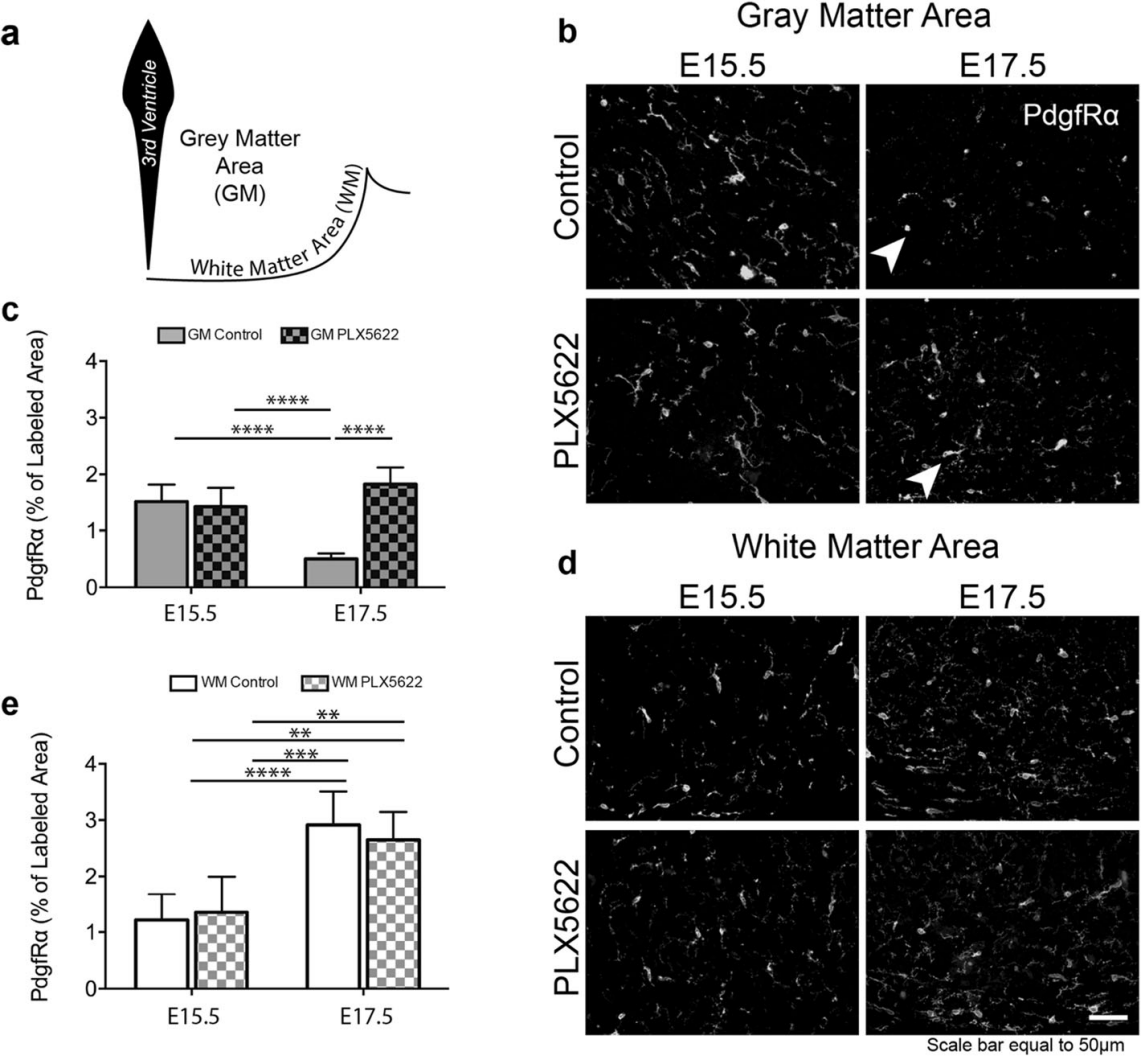
Microglia influence the phenotype of PdgfRα+ late embryonic OPCs in the tuberal hypothalamus. **a** Illustrative diagram depicting the GM and WM areas from which PdgfRα immunolabeled sample bins were set to capture percent area coverage calculations. **b** Representative PdgfRα immunostained regions from control and PLX5622 treated embryonic coronal brain slices from the GM area at E15.5 and E17.5. Arrows indicate examples of PdgfRα+ cells that appear amoeboid in the control but ramified in the PLX5622 treated. **c** Percent area coverage of PdgfRα+ cells from control and PLX5622 treated embryonic coronal brain slices at E15.5 and E17.5 from the GM area (ANOVA, *P* < 0.0001). **d** Representative PdgfRα immunostained regions from control and PLX5622 treated embryonic coronal brain slices from the WM area at E15.5 and E17.5. **e** Percent area coverage of PdgfRα+ cells from control and PLX5622 treated embryonic coronal brain slices at E15.5 and E17.5 from the WM area (ANOVA, *P* < 0.0001). Samples: *n* = 5, 5 embryo brains for control and PLX5622 respectively for both E15.5 and E17.5 time points. Graphs: Bar graph with mean ± SD. Statistics: ANOVA with Tukey post hoc; **P ≤ 0.01, ***P ≤ 0.001, ****P ≤ 0.0001

To determine whether a delay in migration of these glial precursors had any lasting effects on oligodendrocyte maturation, we quantified the morphology of PdgfRα+ cells in both the grey matter (GM), where neuronal cell bodies reside, and white matter (WM) regions, where early axonal tracts are developing [60] at E15.5 and E17.5. PdgfRα is expressed in OPCs during the migration and proliferation phase of oligodendrocyte maturation [61–63]. The GM and WM areas are illustrated in Fig. 6a. Given that waves of OPC production are required to fuel the generation of oligodendrocytes, we reasoned that a defect in an embryonic glial precursor wave might affect development of OPCs downstream. We used PDGFR+ OPC process extension as a proxy for maturation since OPC motility and migration, influenced in part by PDGF signaling, must occur prior to maturation and myelination [64].

At E15.5 in control hypothalami, PdgfRα+ cells had a large number of processes and appeared to have a “migratory-type” phenotype in the GM, which was not affected by the absence of microglia (Fig. 6b). Quantification of the percent coverage area of PdgfRα immunostaining in the GM demonstrated equal PdgfRα+ levels between control and PLX5622-exposed brains at E15.5 (Fig. 6c). By E17.5, a time point directly after when microglia congregated at the ventricle, the control PdgfRα+ cells of the GM appeared to take on an amoeboid phenotype with few extended processes, suggesting this initial wave of migration might be slowing, but in the PLX5622-exposed brains the PdgfRα+ cells instead maintained their extended processes, consistent with the phenotype observed at E15.5 (Fig. 6b, arrows). Moreover, by comparing the percent of PdgfRα covered area in a region of the GM in both control and PLX5622, we found that the control sample region had a significant decrease in area coverage of PdgfRα from E15.5 to E17.5 (Fig. 6c). These findings suggest that in the absence of microglia, a less matured “migratory-like” phenotype of PdgfRα+ OPCs persists.

In contrast, the WM regions showed no difference in the percent area coverage of PdgfRα in age-matched sample regions from both control and PLX5622, with both groups demonstrating an equal increase in percent area coverage of PdgfRα+ from E15.5 to E17.5 (Fig. 6d, e). The increase in percent area coverage of PdgfRα in the WM as the embryo ages is unsurprising since PdgfRα+ OPCs often migrate to axonal tracts where they will eventually begin to myelinate [65].

### Microglia have no effect on early astrocytogenesis in the tuberal hypothalamus

**Fig. 7:**
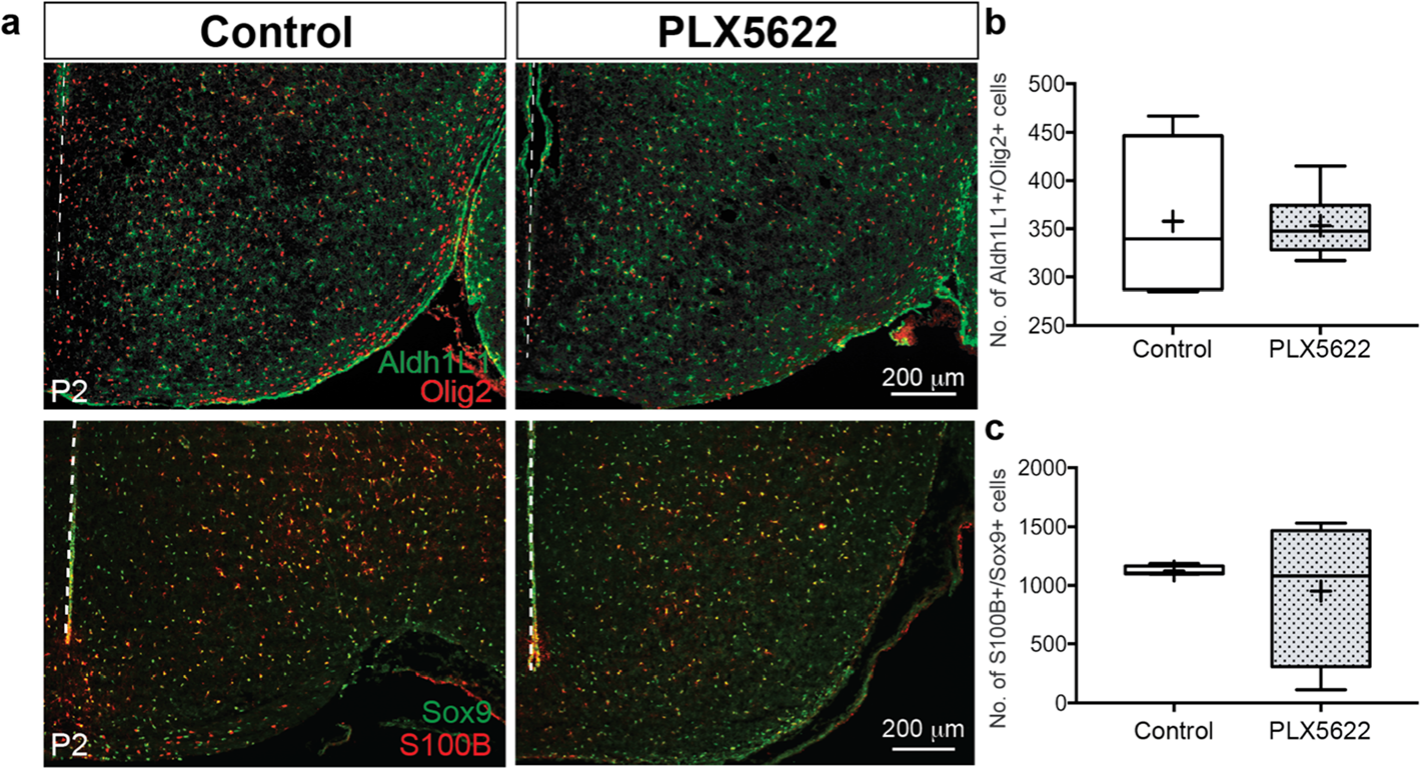
Microglia do not influence the number of early intermediate developing astrocyte populations in the tuberal hypothalamus. **a** Representation of Olig2, Aldh1L1, Sox9, and S100β immunostaining from control and PLX5622 treated P2 brains. Third ventricle outlined with dotted white line. **b** Aldh1L1+/Olig2+ cell counts (*P* = 0.9939) and **c** S100β+/Sox9+ cell counts (*P* = 0.5662) in the tuberal hypothalamus from control and PLX5622 treated P2 brains. Samples: Counts combined from three brain slices in *n* = 5, 6 in Aldh1L1+/Olig2+ counts and *n* = 4, 4 for S100β+/Sox9+ counts for control and PLX5622 brains, respectively. Graphs: Box and Whisker plots with middle line representing median, cross representing mean, box extending from the 25th to 75th percentiles, and whiskers at max and min values. Statistics: Student’s *t* test

Given that astrocytogenesis initiates in the tuberal hypothalamus at E15.5 and that a small subset of Olig2+ cells are known to give rise to astrocytes [57, 58, 66], we next determined if this delay in Olig2+ glioblast migration influenced astrocytogenesis. Aldh1L1 is an astrocyte marker [67] with expression in developing astrocytes from birth through the intermediate astrocyte phase and into adulthood [68, 69]. We examined the total number of Aldh1L1+ astrocytes co-labeled with Olig2+ in the tuberal hypothalamus in the presence and absence of microglia (e.g., following PLX5622 exposure) at postnatal day 2 (P2), a time point after which the radial glia-type neural progenitor pool has been depleted and markers for astrocytes become expressed. Since Aldh1L1 is a cytoplasmic marker, we confirmed each Aldh1L1+ cell nucleus with DAPI and Olig2 immunostaining. Interestingly, we found that all Aldh1L1+ cells co-labeled with Olig2 at this P2 time point. We observed no difference in the number of Aldh1L1+/Olig2+ cells between PLX5622 microglia knockdown and control brains in early postnatal stages (Fig. 7a, b). We also quantified developing astrocytes using another well-known glial marker, S100B [70, 71]. We co-labeled with the glial marker, Sox9, because although S100B is a distinct astrocyte marker in adult brains, it also can be highly expressed in OPCs committed to differentiate into oligodendrocytes in embryonic and postnatal development [72, 73] as well as a small subset of postnatal neurons [74]. Sox9 is expressed in glioblast populations [35, 53], but its expression is downregulated in maturing oligodendrocytes and persists permanently in astrocytes, allowing it to be used as a maturing astrocytic marker [53, 75, 76]. Thus, we quantified S100B+/Sox9+ astrocytes, using DAPI to confirm cell nuclei. We observed no difference in the numbers of S100B+/Sox9+ cells in the absence of microglia when compared to control (Fig. 7a, c). Overall, this suggests microglia do not influence the number of early intermediate embryonic astrocyte progenitor cells.

### Downregulation of cytokines in embryonic slice culture lacking microglia influences proliferation of hypothalamic neurospheres

**Fig. 8:**
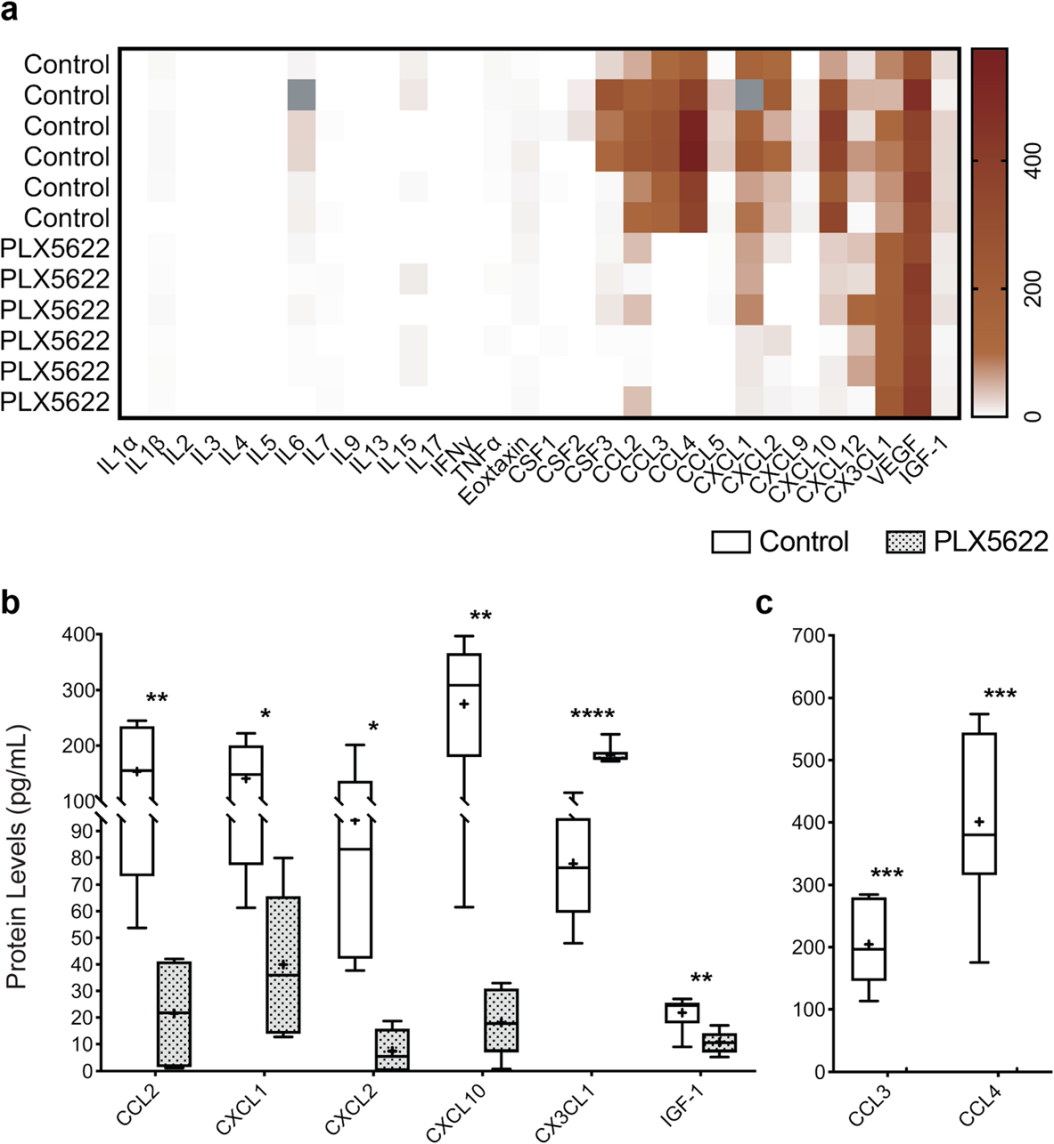
Cytokine profile of slice culture media taken from control and PLX5622 embryonic mouse brain slices of the tuberal hypothalamus. **a** Heatmap of an array of cytokine, chemokine, and growth factor protein levels (pg/mL) from slice culture media. Samples marked with grey boxes were removed as being statistical outliers when analyzed with Dixon’s Q test with 95% confidence. Select chemokines **b** CCL2 (*P*= 0.0085), CXCL1 (*P* = 0.0211), CXCL2 (*P* = 0.0193), CXCL10 (*P* = 0.0034), CX3CL1 (*P* ≤ 0.0001), IGF-1 (*P* = 0.0077), **c** CCL3 (*P* = 0.0008), and CCL4 (*P* = 0.0010) found to be significantly different between control and PLX5622 treated samples. Samples: Culture media samples from *n* = 6 brain slices from separate embryos for both control and PLX5622, except in CXCL1 where *n* = 5 for control. Graphs: Box and Whisker plots with middle line representing median, cross representing mean, box extending from the 25th to 75th percentiles, and whiskers at max and min values. Statistics: Welch’s *t* test, *P ≤ 0.05, **P ≤ 0.01, ***P ≤ 0.001, ****P ≤ 0.0001

Given that cytokines and growth factors play key roles in influencing NPC proliferation and differentiation, as well as glial cell differentiation and migration, we next examined whether our observed microglia influence on gliogenesis was via cytokine/chemokine signaling. We employed PLX5622 to knock down microglia in the developing embryonic brain and compared the levels of growth factors and cytokines +/− microglia. Specifically, we harvested and sectioned E14.5 control and PLX5622-treated brains and grew ex vivo tissue cultures of brain slices containing the tuberal hypothalamic area. After 2 days in culture, we analyzed cytokine and growth factor protein levels found in the conditioned culture media from both control and PLX5622-treated slice cultures (Fig. 8a). We measured that CCL2 (MCP-1), CCL3 (MIP1α), CCL4 (MIP1β), CXCL1 (KC), CXCL2 (MIP2), CXCL10 (IP10), and IGF1 all had significant decreases in expression in conditioned media from the PLX5622 treated samples when compared to the control (Fig. 8b, c). We also found that CX3CL1 (Fractalkine) displayed a significant increase (Fig. 8b). Notably, concentrations of CCL3 and CCL4 were decreased to levels below the capability range of our measuring assay to essentially 0 pg/mL (Fig. 8c).

**Fig. 9:**
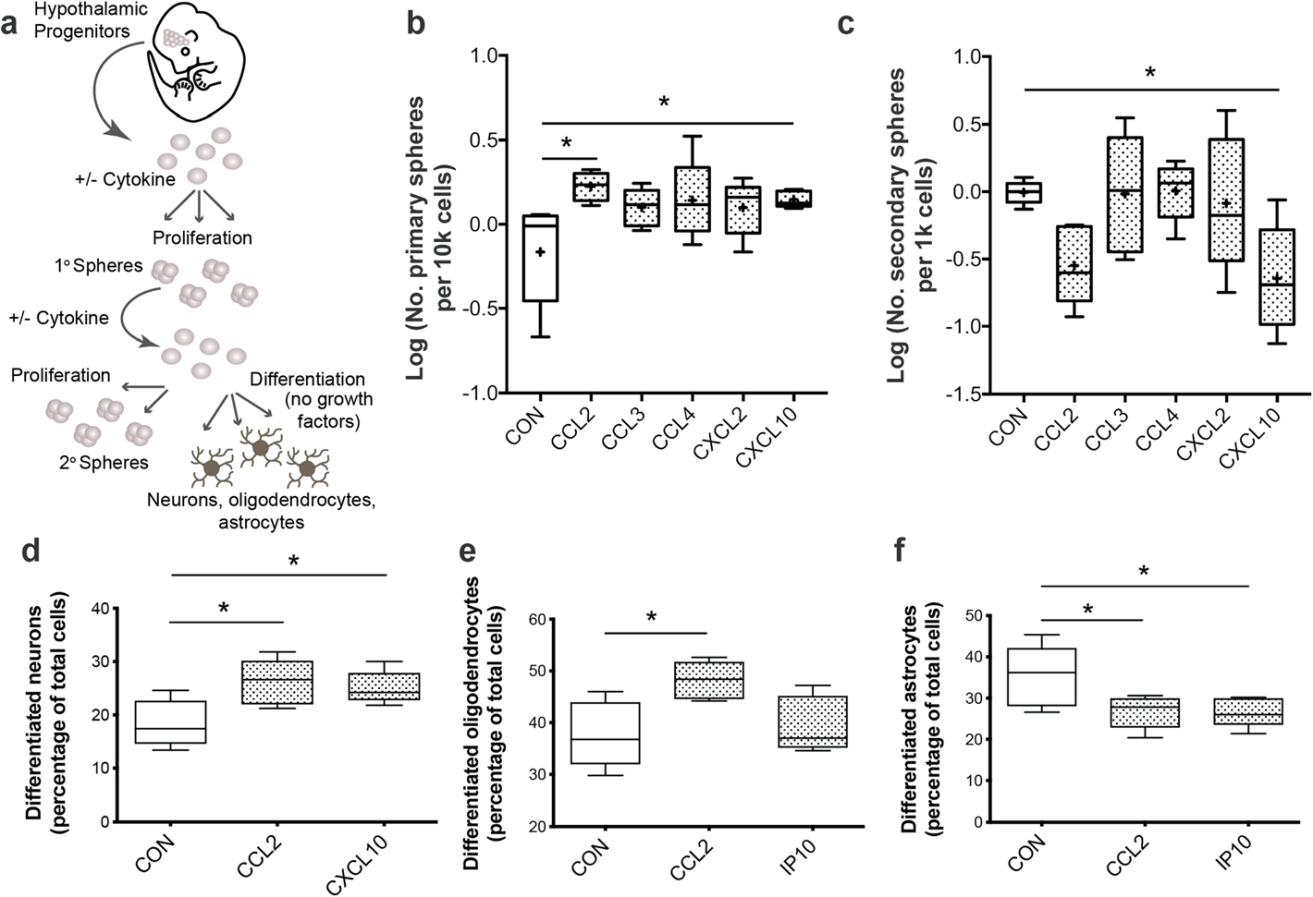
Influence of cytokines on proliferation and differentiation of hypothalamic-derived embryonic NPCs grown in a neurosphere assay. **a** Cartoon depicting the neurosphere proliferation assay. NPCs isolated from the E12.5 embryonic mouse hypothalamus, plated, and left to proliferate. Neurospheres were counted (primary) from plating of 5000 cells/well. These cells were passaged at 1000 cells/well and left to proliferate into secondary neurospheres, or growth factors were removed to allow differentiation into neural lineages. Neurosphere assay of hypothalamic NPCs exposed to cytokines CCL2, CCL3, CCL4, CXCL2, or CXCL10 and assessed for alterations in proliferation ability when compared to control. **b** Primary neurosphere counts from direct plating of embryonic hypothalamic NPCs (ANOVA, *P* = 0.0464). **c** Secondary neurosphere counts following passage from primary neurospheres (ANOVA, *P* = 0.0147). Differentiation assay of hypothalamic neurospheres exposed to CCL2 and CXCL10 then assessed for differentiation into **d** NeuN+ neurons (*P* = 0.023 for CCL2, *P* = 0.048 for CXCL10), **e** PdgfRα+ OPCs (*P* = 0.022 for CCL2), and **f** Aldh1L1+ astrocytes (*P* = 0.44 for CCL2, *P* = 0.041 for CXCL10) when compared to control samples. Samples: Values are represented as number of neurospheres per 5000 cells plated for primary and per 1000 cells plated for secondary (*n* = 5 wells each for control and cytokines extracted from *n* = 5 independent pools of embryonic mouse brains). Values were normalized as fold-change to control and log transformed. Graphs: Box and Whisker plots with middle line representing median, cross representing mean, box extending from the 25th to 75th percentiles, and whiskers at max and min values. Statistics: ANOVA with Dunnet post hoc testing. *P ≤ 0.05

To test the effects of these measured cytokines on NPCs, we treated hypothalamic-derived embryonic NPCs with a subset of cytokines altered in our PLX5622 slice culture, namely CCL2, CCL3, CCL4, CXCL2, and CXCL10, and analyzed their proliferative properties using a neurosphere assay (Fig. 9a). Treatment with CCL2 and CXCL10 increased the number of primary neurospheres colonized from hypothalamic embryonic NPCs (Fig. 9b), demonstrating the potential for these ligands to increase proliferation of hypothalamic NPCs. In contrast, after dissociation and passage of primary neurospheres into secondary neurospheres, CXCL10 treatment caused a decrease in the number of spheres, suggesting that cytokine treatment directed these NPCs towards a differentiated lineage as opposed to an expansive, self-renewing fate (Fig. 9c). CCL2-treated secondary neurospheres were statistically downregulated following a *t* test on control versus CCL2-treated (*P* < 0.01), but this significance was lost when conducting an ANOVA with Dunnet post hoc (*P* = 0.0874).

To determine if CCL2 and/or CXCL10 induced a differentiated lineage, we next quantified the relative numbers of differentiated neurons (e.g., NeuN+), oligodendrocytes (e.g., Pdgfrα+), and astrocytes (e.g., Aldh1L1+) observed in treated and control spheres. We measured an increase in the number of differentiated neurons (Fig. 9d) and oligodendrocytes (Fig. 9e, CCL2 only) but a decrease in astrocytes (Fig. 9f), consistent with these ligands inducing a differentiated state.

## Discussion

Here, we demonstrated a new role for microglia in influencing gliogenesis in the developing embryonic hypothalamus. We showed that by E11.5, microglia invade the tuberal hypothalamus, and by E15.5 they cluster alongside progenitors that lie ventral to the hypothalamic sulcus and adjacent to the third ventricle. Upon this clustering, these ventricular-residing microglia acquire an activated phenotype relative to peripheral microglia located in the hypothalamic mantle zone and are positioned to influence gliogenesis, which is actively occurring at a time and from the same domain against which these microglia cluster. Studies have shown that microglia in the forebrain subventricular zone take on a similar activated form during neurogenesis and gliogenesis [27].

Using a PLX5622 microglia knock down model, we showed that microglia affect tuberal hypothalamic gliogenesis. When microglia are absent, we see a delay in Olig2+ glioblast migration away from the ventricular zone and a concomitant disruption of maturation and migration of OPCs residing in the grey matter at embryonic and early postnatal time points. An analogous in vitro study shows that microglia-conditioned media enhances OPC maturation and myelination without affecting proliferation [77]. We further showed that a subset of cytokines was alternately expressed in embryonic hypothalamic slice cultures in the absence of microglia and that CCL2 and CXCL10 altered the renewal capacity of cultured hypothalamic progenitor cells and drove differentiation of hypothalamic NPCs. In particular, we found that CCL2 and CXCL10 influenced neuronal differentiation at the expense of astrocyte differentiation, with CCL2 further promoting an oligodendrocyte fate. These findings demonstrate that embryonic microglia influence nearby glial precursors through cytokine signaling and are required for proper maturation of oligodendrocytes in the developing tuberal hypothalamus.

In line with our study, previous work has shown that co-culturing of activated microglia or individual cytokines with NPCs can drive differentiation into neurons, oligodendrocytes, and astrocytes, demonstrating that the release of signaling molecules in vitro is sufficient to influence differentiation programs [27–30, 78–80]. For example, CXCL1 can inhibit and arrest oligodendrocyte migration in a concentration dependant manner [81]. Interestingly, this corresponds to our data here that shows a decrease in the amount CXCL1 protein in media from embryonic hypothalamic cultures lacking microglia and a corresponding in vivo increase in a migratory OPC phenotype in the absence of microglia. Moreover, CCL2 can affect adult NPCs in vitro by promoting neuronal differentiation [80], similar to our data herein, whereas IGF-1 can influence NPC differentiation capacity [30] and CCL3 can promote GFAP+ astroglial differentiation of cultured adult NPCs [78]. Although CCL3 did not significantly influence hypothalamic embryonic NPC renewal in our neurosphere assay, CCL3 protein levels were decreased in our embryonic brain slice culture following in utero exposure to PLX5622, suggesting that this cytokine might influence NPCs in vivo. A persistent challenge for the field is the inability to determine in vivo and in real time the effect of individual signaling molecules released by microglia on NPC developmental programs within the endogenous signaling milieu. Given the importance of the ventricular microenvironment on both NPCs and microglia behaviors, ex vivo slice cultures likely fail to capture the true signaling situation.

A key finding herein is that these ventricular-residing microglia likely influence nearby NPC behavior via exocrine signaling. Initially, we explored whether microglia phagocytosed their neighboring NPCs, as has been previously observed in the embryonic cortex [22]. We failed to observe any overlap of Olig2/Iba1 or Sox9/Iba1 immunostaining in our imaging, even at higher magnification, that would be suggestive of phagocytosis of cell bodies. Instead, these ventricular microglia appeared to be just lying adjacent to the progenitors. Furthermore, if microglia were indeed involved in the termination of neurogenesis via engulfment of residual NPCs (as per Cunningham et al.) [22], then we would predict an extension of neurogenesis in the absence of microglia. Instead, the elimination of microglia in the tuberal hypothalamus using the CSF1R inhibitor PLX5622 had no effect on the timing or duration of the neurogenic curve in the tuberal hypothalamus (data not shown), suggesting that these ventricular-residing microglia do not have an obvious influence on embryonic neurogenesis.

Despite the lack of evidence for neuronal cell phagocytosis in our experiments, we still observed expression of lysosomal markers, CD68 and LAMP1. We could hypothesize from previous studies that our observed increase in expression of lysosomal proteins is due to engulfment of non-nuclear types of celluar debris such apoptotic synapses [11, 82], accumulated oligomers [83], or extracellular vesicles (EVs) [84]. Likewise, it is possible that degradation of extracellular matricies via podasomes [85], shown to contain lysomal proteins [86], could allow microglia to navigate through and sculpt the developing brain parenchyma that surrounds NPCs during this key window of development when neurogenesis is ending and gliogenesis is commencing. Interestingly, new evidence suggests microglia phagocytosis and NPC-derived EVs are involved in a cascade of signaling events in NPC-microglia cross talk, leading to modulation of the NPC niche [84, 87]. Such mechanisms could be involved in influencing hypothalamic embryonic NPCs whereby bidirectional microglia-NPC signaling occurs. For example, in the cortex, neural stem cells in the SVZ secrete EVs that are engulfed by nearby microglia, which causes them to become more rounded in appearance and migrate closer to the ventricle [84]. These engulfed EVs also elicit cytokine release from the activated microglia that then signal back to the neural stem cells to decrease their renewal capacity [84]. It is tempting to speculate that the ameboid microglia that reside near the ventricle in the embryonic hypothalamus might be receiving EV signals from the nearby NPCs, which then induces the microglia to release the cytokine/growth factors that we observed in this study. Consistently, treatment of hypothalamic NPCs in culture with these measured cytokines caused a reduced renewal capacity and an ultimate push of these NPCs towards a fate-committing pathway, further suggesting that this “serve-and-return” between neural stem cells and microglia might also exist in the embryonic hypothalamus. Moreover, recent work has suggested that secretomes derived from phagocytic microglia provide necessary signaling for the proper maintenance and survival of adult hippocampal NPCs [87], adding complexity to the mechanisms of NPC-microglia cross talk. In summation, it appears that microglia and NPCs maintain an intricate balance of bidirectional communication which simultaneously influences the behavior of both cell types.

Curiously, we saw an increase in only one cytokine, CX3CL1, in our microglia knock down model. CX3CL1 is thought to be mainly expressed by neurons and astrocytes. The sole receptor, CX3CR1, is highly expressed on the surface of microglia but also at low levels on OPCs. It is involved in regulating a broad spectrum of microglia properties including chemotaxis, neuronal synapse formation, and has been shown to promote OPC differentiation, although not proliferation [88–91]. We suspect upregulation of CX3CL1, seen in our microglia knock down model, occurs when the developing hypothalamic cells are faced with a lack of reciprocal interaction between CX3CL1 and CX3CR1 in the absence of microglia. We predict upregulation is an attempt to recruit these absent microglia during this key phase of oligodendrocyte development, although additional studies are needed.

A limitation of this study is the paucity of glial markers that distinctly label maturing oligodendrocytes from astrocytes. Indeed, aside from its well-known role in oligodendrocyte development, *olig* genes are underappreciated for their ability to also drive specification of subsets of astrocytes. Consistent with the notion that Olig2 serves a role in pan-glial cell fates, lineage tracing studies in the spinal cord show a spatially restricted ventricular zone (VZ) Olig2+ domain—similar to that observed in the tuberal hypothalamus at E13.5—is responsible for producing ventrally-located GFAP+ astrocytes [56]. Likewise, in the developing SVZ, Olig2 is intimately involved in astrocyte development whereby Olig2 is transiently expressed in nearly all early maturing GFAP+ astrocytes, becoming downregulated once substantial GFAP expression exists during astrocytic differentiation [57], further demonstrating the challenges of separating early oligodendrocytes from astrocytes. In addition, Aldh1L1 is a distinct marker for developing astrocytes and the only astrocyte-lineage gene known to be expressed in the embryonic hypothalamus [35], but it labels a wide array of various types of developing astrocytes. This non-specificity makes it difficult to detect specific subsets of developing astrocyte populations that could be affected by the loss of microglia. Indeed, a decrease in astrocyte numbers following the elimination of microglia in vivo might have been observed—and more in alignment with the neurosphere in vitro results—if selective markers for astrocyte subpopulations were available.

## Conclusion

In summary, our results show that microglia can influence glial development in the embryonic hypothalamus and demonstrate that cytokine signaling can further modulate NPC behaviors in culture. Further work is needed to dissect the intricate intracellular pathways that link cytokines signaling with NPC proliferation and differentiation. Our research adds to the growing body of the evidence highlighting the various and diverse roles of microglia during embryonic development.

## Supplementary information

**Additional file 1:** Supplementary Figure 1. Successful knock down of microglia using the Csf1R antagonist, PLX5622, given to dams in chow starting at E3.5 onwards***.*** (a) Magnified images from control of Iba1 and Csf1R to view cell morphology and merged imaging. The majority of Csf1R+ cells co-label with Iba1 with rare exceptions (white arrow) that still appear to have a microglial cell morphology. (b) Timeline of microglia knock down model whereby pregnant dams are given PLX5622 or control diet starting at E3.5 and embryonic/postnatal samples were taken at E15.5, E17.5 and P2. (c) *In situ* hybridization using a *csf1r* probe to identify mRNA expression in P2 tuberal hypothalamic brain slices in PLX5622 treated and control treated animals. Scattered labeling of cells with *csf1r* mRNA is seen in the control with some concentration at the ventricle but expression appears to be absent in the PLX5622 model. There is background staining in the PLX5622 sample, but this does not take on the same robust, and cell-like appearance of positive staining seen in the control sample. (d) Control shows both Iba1+ and Csf1R+ cells with microglia-like morphology that are absent in dams treated with PLX5622, which only shows background blood vessel staining. Scale bar = 100μm.

## Data Availability

All data generated or analyzed during this study are included in the manuscript submission and its supplementary figure.

## Abbreviations

CNS: Central nervous system
*E*: Embryonic day
EV: Extracellular vesicles
GM: Grey matter
NPC: Neural progenitor cell
OPC: Oligodendrocyte progenitor cell
*P*: Postnatal day
PBS: Phosphate buffered saline
SVZ: Subventricular zone
WM: White matter
VZ: Ventricular zone
