# Supplementary figures and images for: Embryonic microglia influence developing hypothalamic glial populations
Source: J Neuroinflammation. 2020 May 6;17:146. doi: 10.1186/s12974-020-01811-7 (PMC7201702; doi:10.1186/s12974-020-01811-7)

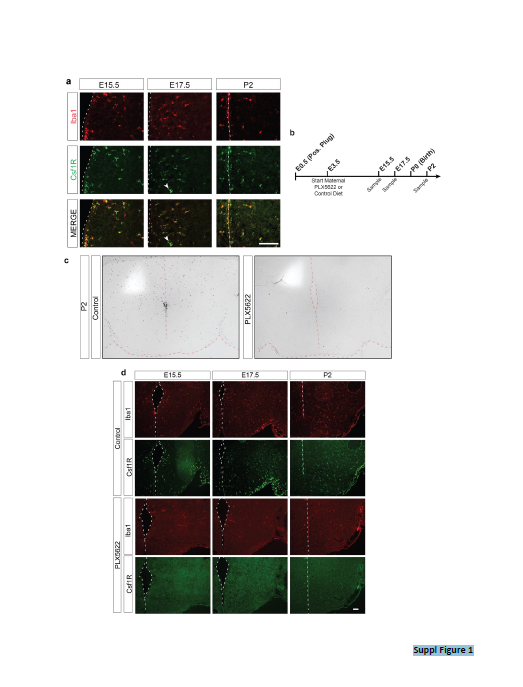

Supplement: Supplementary file 1 — Additional file 1: Supplementary Figure 1. Successful knock down of microglia using the Csf1R antagonist, PLX5622, given to dams in chow starting at E3.5 onwards. (a) Magnified images from control of Iba1 and Csf1R to view cell morphology and merged imaging. The majority of Csf1R+ cells co-label with Iba1 with rare exceptions (white arrow) that still appear to have a microglial cell morphology. (b) Timeline of microglia knock down model whereby pregnant dams are given PLX5622 or control diet starting at E3.5 and embryonic/postnatal samples were taken at E15.5, E17.5 and P2. (c) In situ hybridization using a csf1r probe to identify mRNA expression in P2 tuberal hypothalamic brain slices in PLX5622 treated and control treated animals. Scattered labeling of cells with csf1r mRNA is seen in the control with some concentration at the ventricle but expression appears to be absent in the PLX5622 model. There is background staining in the PLX5622 sample, but this does not take on the same robust, and cell-like appearance of positive staining seen in the control sample. (d) Control shows both Iba1+ and Csf1R+ cells with microglia-like morphology that are absent in dams treated with PLX5622, which only shows background blood vessel staining. Scale bar = 100μm. [file 12974_2020_1811_MOESM1_ESM.docx]
